# Optimisation of Milk Protein Top-Down Sequencing Using In-Source Collision-Induced Dissociation in the Maxis Quadrupole Time-of-Flight Mass Spectrometer

**DOI:** 10.3390/molecules23112777

**Published:** 2018-10-26

**Authors:** Delphine Vincent, Dominik Mertens, Simone Rochfort

**Affiliations:** 1 Department of Economic Development, Jobs, Transport and Resources, AgriBio Centre, Bundoora, Victoria 3083, Australia; simone.rochfort@ecodev.vic.gov.au; 2 Genedata AG, 4016 Basel, Switzerland; Dominik.Mertens@genedata.com; 3 School of Applied Systems Biology, La Trobe University, Bundoora, Victoria 3083, Australia

**Keywords:** top-down proteomics, HPLC-ESI-Q-TOF MS, cow’s milk, whey proteins and caseins, tandem MS

## Abstract

Top-down sequencing in proteomics has come of age owing to continuous progress in LC-MS. With their high resolution and broad mass range, Quadrupole Time-of-Flight (Q-ToF) hybrid mass spectrometers equipped with electrospray ionisation source and tandem MS capability by collision-induced dissociation (CID) can be employed to analyse intact proteins and retrieve primary sequence information. To our knowledge, top-down proteomics methods with Q-ToF have only been evaluated using samples of relatively low complexity. Furthermore, the in-source CID (IS-CID) capability of Q-ToF instruments has been under-utilised. This study aimed at optimising top-down sequencing of intact milk proteins to achieve the greatest sequence coverage possible from samples of increasing complexity, assessed using nine known proteins. Eleven MS/MS methods varying in their IS-CID and conventional CID parameters were tested on individual and mixed protein standards as well as raw milk samples. Top-down sequencing results from the nine most abundant proteoforms of caseins, alpha-lactalbumin and beta-lactoglubulins were compared. Nine MS/MS methods achieved more than 70% sequence coverage overall to distinguish between allelic proteoforms, varying only by one or two amino acids. The optimal methods utilised IS-CID at low energy. This experiment demonstrates the utility of Q-ToF systems for top-down proteomics and that IS-CID could be more frequently employed.

## 1. Introduction

Top-down proteomics, a term invented by Kelleher and colleagues 20 years ago [1], describes the analysis of intact proteins, either in their native form or more often in a denatured state, which allows for a characterisation of proteoforms as comprehensively as possible. Coined in 2014, the term proteoform “designates all of the different molecular forms in which the protein product of a single gene can be found, encompassing all forms of genetic variation, alternative splicing of RNA transcripts, and post-translational modifications (PTMs)” [2]. The analysis of intact proteins is now always performed using mass spectrometry (MS) and technical progresses in top-down proteomics are tightly linked to improvements made on mass analysers. The most important technological advance was the coupling of the soft ionisation technique, electrospray ionisation (ESI), to a mass spectrometer and the production of gas phase ions from large molecules [3], which led to its first application to intact proteins [4,5]. ESI generates multiply-charged protein ions of low *m*/*z*, thereby allowing the analysis of very large molecules, even on an MS platform with a limited mass range. By applying a deconvolution algorithm [6], the resulting complex multi-peak spectrum can be converted into a single peak corresponding to the true molecular weight of the molecule. Further developments in intact protein analysis quickly followed with the introduction of complex multistage mass analysers of high resolution such as linear ion trap (LIT), quadrupole time-of-flight (Q-ToF), Fourier transform (FT) ion cyclotron resonance, and orbitrap instruments. 

Today, various methods of ion fragmentation are available, such as collision-induced dissociation (CID, also called collisionally activated dissociation CAD), in-source CID (IS-CID, also known as up-front CID, cone-voltage CID, and nozzle-skimmer dissociation), higher-energy collisional dissociation (HCD), electron capture dissociation (ECD), and electron transfer dissociation (ETD) (for review [7]). Different types of fragmentation modes yield different information about the structure and composition of the analyte. Tandem mass spectrometers that carry out CID to generate product ions from precursor ions have proven extremely useful for the identification and characterisation of proteins from a complex mixture. CID was first described by McLafferty and Bryce in 1967 [8] and Jennings in 1968 [9]. Parent ions collide with neutral gas atoms or molecules (typically helium, nitrogen or argon), which result in the formation of b- and y-type ions. The efficacy of a CID experiment will depend on the relative translational energy of the ion and target, the nature of the target, the number of collisions that is likely to take place, and the *m*/*z* window of the instrument. Initial top-down sequencing experiments exploited low-energy CID methods to induce protein fragmentation. However, for large molecules, CID does not produce a fragmentation pattern comprehensive enough to fully characterise proteins, but rather produces enough fragments or sequence tags to identify the protein. Additionally, if the protein contains PTMs, low-energy CID most likely will not be sufficient to localise the modified site or the PTM may be the preferred site of cleavage [10,11]. IS-CID is a proven albeit seldom utilised fragmentation mode allowing single-stage instrumentation, such as single quadrupole (Q) or ToF mass analysers, to produce spectra similar to those obtained with far more expensive hybrid instruments. In this method, invented by Katta and colleagues in 1991 [12], CID is carried out within the ion source in the high-pressure region between the capillary exit and the skimmer entrance to the Q mass spectrometer. Because there is no prior selection of the precursor ion, this does not qualify as a bona fide MS/MS experiment. By manipulating lens voltages that channel ions from the source to the mass analyser, relatively low-energy ions formed in the atmospheric pressure region of the source collide with residual background gas, usually nitrogen, in the transition region. As a result, excited ions can undergo unimolecular decomposition to produce fragment ions. As for CID, only b- and y-type ions are observed. Tandem spectra from protonated peptides produced by IS-CID or low-energy CID are comparable [13,14]. In-source dissociation has also been demonstrated for whole protein ions [15]. The time scale for IS-CID is on the order of a few hundred microseconds to a few milliseconds, which is much faster than that of CID. IS-CID fragmentation can be reproducible provided ion source parameters, such as temperature, pressure, voltage, and sample purity are tightly controlled [16]. 

Kelleher in 2004 [17] rigorously defines top-down proteomics as a multistep process whereby the molecular weights (MWs) of intact proteoforms are accurately measured using a high-resolution mass analyser in combination with a direct fragmentation of the protein ions using tandem MS. Top-down sequencing of intact proteins were initially performed using triple quadrupole (QQQ) instruments and IS-CID [5]. In 1990, Loo and colleagues characterised a 14-kDa bovine ribonuclease A in its native and reduced forms using CID [18]. Soon after, higher mass resolution was achieved by applying FT-MS to equine cytochrome c, porcine albumin, thioredoxin and ubiquitin [19], myoglobin [20], and carbonic anhydrase [15]. A hybrid Q-ToF instrument, also called Q-ToF [21], was invented in 1996 by Morris and colleagues [22]. Q-ToF mass spectrometers combine the quadrupole one (Q1) in which ion precursors are funnelled through and selected, the quadrupole collision cell (q) of a QQQ in which selected precursor ions are fragmented usually through CID and more recently ETD, and a reflector ToF detector in which the fragment ions are mass analysed. Spectra obtained in both full-scan (MS1) and MS/MS (MS2) modes exhibit clean spectra due to orthogonal pulsing into the ToF section, high mass accuracy along with stable isotopic resolution across the *m*/*z* range, and high fidelity meaning accurate isotopic ratios. This permits the determination of charge states and unambiguous assignment of the mono-isotopic signal of the intact molecules. This leads to accurate prediction of AA sequence and successful identification of peptides and proteins of medium MW via database searches by tightening the search parameters and augmenting the confidence in the results. Q-ToF instruments perform well for quantitative analyses [23] and for the identification of PTMs [24]. Q-ToF were demonstrated to outperform QQQ mass spectrometers owing to both their enhanced sensitivity and resolution in the region of one to two orders of magnitude [22]. This initial observation was further validated on phosphopeptides; the higher resolving power of the Q-ToF improved the selectivity and sensitivity of parent ions, thus minimising interference from other product ions and maximising sequencing results [25]. 

The first use of LC-ESI-Q-ToF MS in top-down sequencing of intact proteins was reported by Nemeth-Cawley and Rouse in 2002 [26], who used CID to fragment ion precursors from eight known protein standards, ranging from 5 to 66 kDa. Peptide sequence tags thus obtained led to the unambiguous identification of the analysed proteins, along with the characterisation of disulphide bonds and glycosylation. While their method was successful, the authors noted some limitations: first, it could not yet be applied to complex protein mixtures without prior separation; secondly, at a low concentration, the signal-to-noise ratio was affected, thus necessitating longer MS/MS acquisition times. Based on their experience, the following year, the same group applied their validated method to characterise a recombinant immunoglobulin gamma-1 (IgG-1) fusion protein [27]. The top-down strategy allowed them to demonstrate that the recombinant protein was expressed as a full-length form as well as N- and C-processed truncated proteoforms [27]. In 2004, Ginter and colleagues exploited both IS-CID and traditional CID mode of a Q-ToF mass spectrometer, thereby achieving pseudo-MS3 levels, to top-down sequence seven known proteins spanning from 11 to 66 kDa [28]. Individual sequence tags of 10 to 26 AAs were retrieved from both N- and C-termini, and unambiguous identification of protein standards was achieved. IS-CID on its own was used to identify various purified recombinant proteins using ESI-Q-ToF MS [29]; fragmentation efficiency depended on cone voltage and y-ions formed predominantly by cleavage on the C-terminal side of nonpolar residues. An unknown 4.9 kDa recombinant peptide was completely sequenced using LC-ESI-Q-ToF MS/MS of 10,000 resolution and traditional CID fragmentation; complete y- and b-type ion series were obtained and the formation of beta-mercaptoethanol adducts was reported [30]. In 2009 Armirotti and colleagues reported 90–100% sequence coverage of horse myoglobin (17 kDa) and bovine carbonic anhydrase II (30 kDa) using LC-ESI-Q-ToF MS/MS and CID fragmentation mode [31]. They could also identify an unknown protein as superoxide dismutase (16 kDa) and locate one acetylation site. ESI CID Q-TOF MS/MS was used to identify eight intact antimicrobial peptides from Asian frog skin and locate disulphide bridges [32]. ESI-Q-ToF technology has also been employed to investigate the precise stoichiometry of protein assemblies, the interactions between subunits and the position of subunits within the complex [33]. 

In our laboratory, we host a maXis HD UHR-Q-ToF (60,000 resolution) with an ESI source on-line with a UHPLC 1290 Infinity Binary LC system (Agilent, Mulgrave, VIC, Australia), which we have used to develop a top-down method to analyse cow’s milk proteins [23]. This method was further implemented with CID MS/MS analysis and applied to the study of UHT milk shelf life [34]. The present study aimed at optimising MS/MS analysis by testing the effect of some of the parameters pertaining to IS-CID in combination or not with conventional CID. A total of 11 MS/MS methods were assessed on samples bearing increased complexity, namely individual milk protein standards, mixed protein standards and cow’s raw milk samples from Jersey or Holstein breeds. Milk proteins exhibit numerous PTMs, however in this initial study, we focused on optimising protein fragmentation to achieve the greatest sequence coverage possible. To this end, nine of the most abundant proteoforms of milk caseins and whey proteins, including genetic variants, were compared across samples and MS/MS methods whose efficacy was assessed based on protein sequence coverage alone. This necessitated developing processing workflows using the powerful Genedata Expressionist software to thoroughly explore and annotate the MS1 and MS2 files.

## 2. Results and Discussion

### 2.1. Sample Complexity

**Figure 1:**
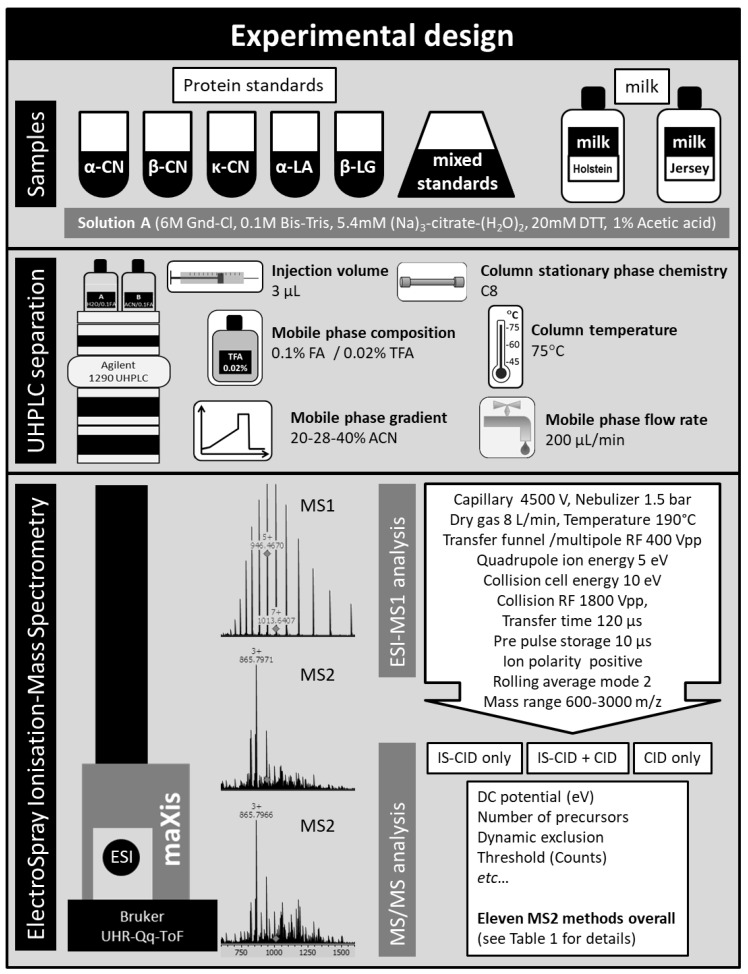
Schematic diagram of the experimental design of the study.

In this experiment, we wanted to test whether sample complexity would impact the quality of spectral acquisition. To this end, we prepared a set of samples displaying increasing proteome complexity: from simple individual milk protein standards, albeit varying in their purity level from 70% (α-CN) to 98% (β-CN), to a mixture of these standards, to the highly complex biological matrices raw milk samples that were obtained from two common cow breeds. Figure 1 describes the experimental design of this study. 

#### 2.1.1. UPLC-ESI-Q-ToF MS Can Handle Complex Samples

**Figure 2:**
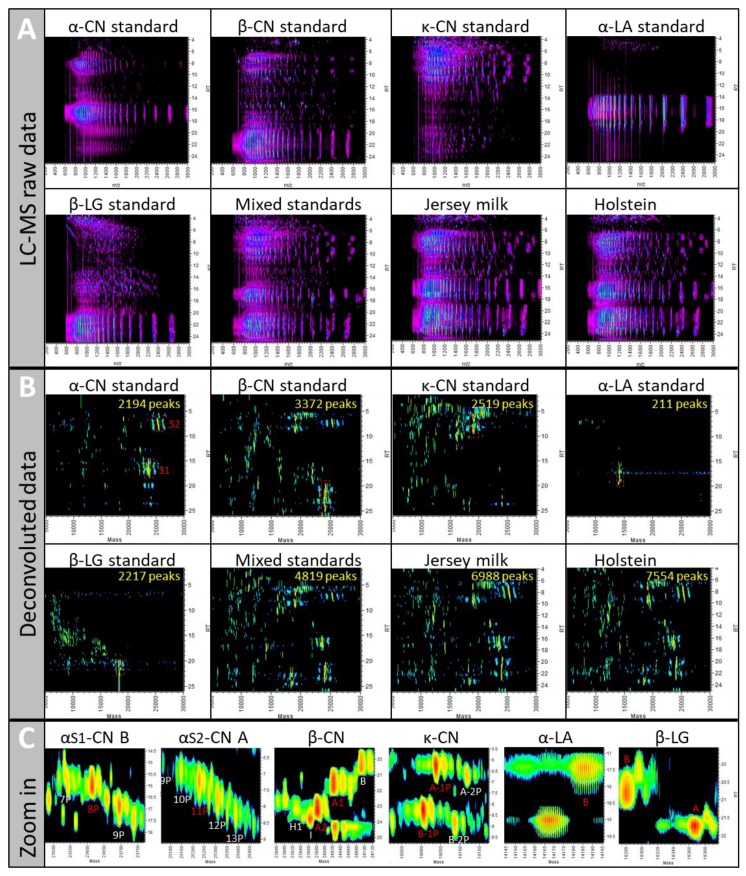
LC-MS maps of the different samples (individual protein standards, mixed protein standards, Jersey milk, and Holstein milk) visualised in Genedata Refiner pre- and post-deconvolution with *m*/*z* on the x axis and LC retention time in min on the y axis. (**A**) LC-MS1 unprocessed raw data. (**B**) Deconvoluted data displaying proteins of interest in the red boxed areas. The total numbers of deconvoluted peaks detected are indicated in yellow. (**C**) Close-ups of the boxed areas of the individual protein standards; the nine proteoforms highlighted in red are the proteins targeted for method validation.

While the raw data clearly illustrate increasing complexity with more LC-MS1 peaks detected in the milk samples compared to the individual protein standard samples (Figure 2A), the latter are more complex than first anticipated, particularly the β-CN standard, which is claimed to be 98% pure. 

These standards were analysed using a bottom-up shotgun approach and yielded many protein hits [35]. The α-LA standard sample (85% pure) generated by far the simplest LC-MS1 pattern, with clear elution from 14 to 19 min and distinct *m*/*z* peaks of the charged envelope (Figure 2A). 

The LC-MS1 pattern of the mixed standard sample is quite similar and as complex as the patterns of milk samples owing to the fact that the individual protein standards are far from pure. Indeed, following protein mass deconvolution and the display of protein accurate masses separated by HPLC (Figure 2B), many deconvoluted peaks appear besides the expected proteins of interest. Such a display confirms that the α-LA standard is the least complex sample with only 211 deconvoluted peaks, whereas the other individual standards display between 2194 (α-CN) and 3372 peaks (β-CN). 

With 4819 deconvoluted peaks, the mixed standard sample is indeed of intermediate complexity between the individual standards and the raw milk samples, which resolved 6988 and 7554 peaks in Jersey and Holstein cows, respectively. While proteins displayed some degree of co-elution during the HPLC separation, particularly in complex samples, separation along the *m*/*z* range could isotopically resolve all proteins of less than 30 kDa, thus yielding deconvoluted monoisotopic masses (also shown in [23]). 

#### 2.1.2. The 11 MS/MS Methods Are Assessed Using a Reference Set of Known Proteins

Another aim of our study was to optimise the MS/MS method by modifying key parameters and devise objective tools to compare the different methods. We thought the best comparative approach would be a targeted one relying on a small reference set of known proteins. The proteins of interest are depicted in the boxed areas of Figure 2B which have been zoomed in in Figure 2C. We chose nine prominent proteoforms, some of them displaying allelic variations, in each of the individual standards, namely, α-CN type S1 variant B with 8 phosphorylations (α_S1_-CN B-8P, 23,600.3 Da), α-CN type S2 variant A with 11 phosphorylations (α_S2_-CN A-11P, 25,213.0 Da), β-CN variants A1 and A2 (β-CN A1 24,008.2 Da, β-CN A2 23,968.2 Da) each with five phosphorylations, κ-CN variants A and B with 1 phosphorylation (κ-CN A-1P 19,026.5 Da, κ-CN B-1P 18,993.6 Da), α-LA variant B (α-LA B 14,176.8 Da), and β-LG variants A and B (β-LG A 18,355.5 Da, β-LG B 18,269.4 Da). We have previously used these proteins to optimise a quantitative LC-MS method [23]. The full primary sequence of these proteins is known, and therefore fragmentation efficiency for top-down sequencing purpose could be assessed. This is presented in the last chapter. While not included in this article, our MS/MS method also allowed us to identify various PTMs of milk most abundant proteins such as lactosylation (+324 Da), oxidation (+16 Da), glycosylation ([Hex(1)HexNAc(1)NeuAc(2)] + 947 Da), and degradation products, as reported in [34].

### 2.2. Effect of IS-CID and CID on Their Own or Combined on Spectral Data

In a recent study [34], we have used conventional CID (Method 2 in the present study) to identify the degradation products of milk’s most abundant proteins occurring following UHT treatment and storage on the shelf. While this top-down sequencing method proved successful for the identification of small proteins and degradation products, we wanted to optimise it for the analysis of intact milk proteins. Our Q-ToF mass spectrometer allows us to perform both IS-CID and CID in a single experiment. The benefits of both have been stated in the introduction of this manuscript. In the present study, we have only slightly varied the parameters controlling CID fragmentation, choosing to operate it in a fully automated manner. We have mostly fine-tuned the parameters pertaining to IS-CID. We thus tested different energy values for IS-CID MS (ion funnel 1 exit) and IS-CID MS/MS (ion funnel 2 entrance) parameters. A total of 11 MS/MS methods were thus devised that employed either CID or IS-CID on their own or combined (Table 1). 

In summary, Methods 2 and 3 employed CID only, Methods 4 and 12 employed IS-CID only, and Methods 5–11 employed IS-CID within the ESI source/ion transfer region, followed by CID within the collision cell. 

**Figure 3:**
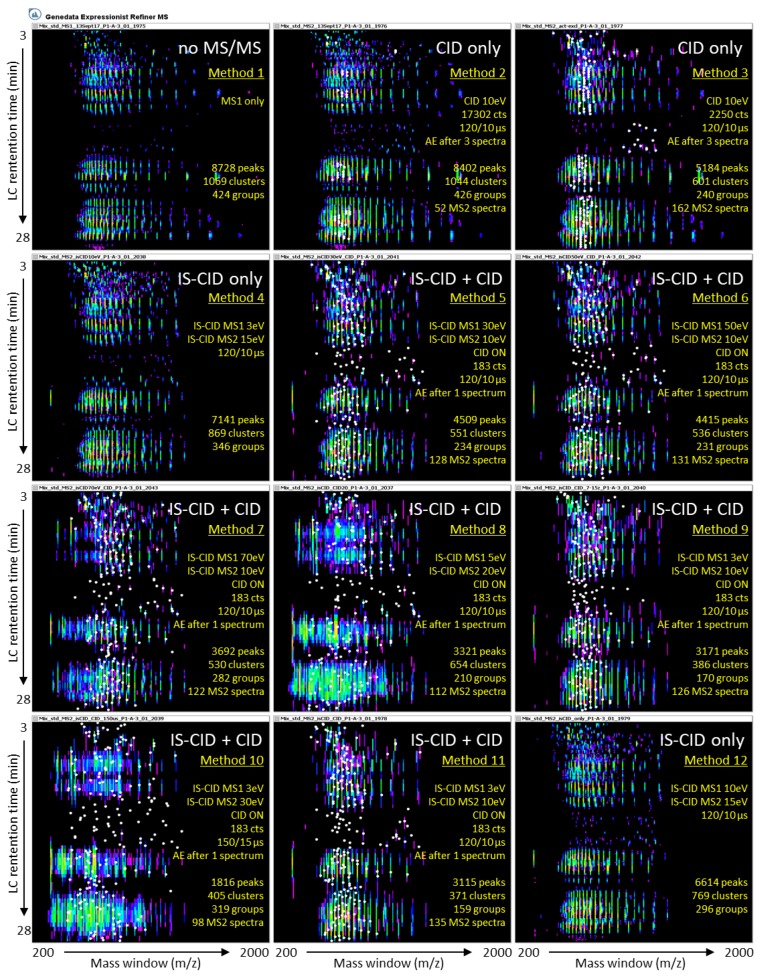
LC-MS maps of the mixed protein standards sample visualised in Genedata Refiner with the scanned mass range (200–2000 *m*/*z*) on the x axis and LC retention time (3–28 min) on the y axis across all 12 methods following data processing. White dots represent true MS/MS (MS2) events using conventional CID fragmentation. AE, active exclusion.

Figure 3 displays the LC-MS patterns for each of the 12 methods using the mixed protein standards sample.

Table 2 compares all 12 methods based on LC-MS and LC-MS/MS observations 

#### 2.2.1. Precursor Intensity Threshold Is a Key Parameter for CID Fragmentation

Methods 2 and 3 (CID only) only differed in the intensity threshold for precursor selection, being almost 7 times higher in Method 2 (2550 counts) relative to Method 3 (17,302 counts) (Table 1). This allows precursor ions of low signal intensity to undergo CID fragmentation. As expected, this did not affect the LC-MS1 data; however, it did result in many more MS/MS events when Method 3 was used. If we consider the mixed standard sample as an example, Method 2 resulted in 8402 ions with only 56 (0.7%) MS2 spectra, whereas Method 3 yielded 5184 peaks and 170 (3.3%) MS2 spectra (Table 2). Consequently, the top-down sequencing efficiency varied between Methods 2 and 3, as will be discussed in the following section. 

Charge state preference aside, in this study we did not attempt to optimise CID conditions and took full advantage of the automatic MS/MS mode available on our instrument with default parameters as it proved efficient [34]. Using CID on its own, Nemeth-Cawley and colleagues successfully top-down sequenced unknown intact proteins purified by affinity and size-exclusion chromatography from mammalian cells using an ESI-Q-ToF MS/MS strategy [27]. 

#### 2.2.2. Ion Funnel 1 Energy Has Little Impact on IS-CID Fragmentation

Methods 4 and 12 (IS-CID only) differed only in the energy applied to the ion funnel 1 exit during the IS-CID MS1 step. Method 4 applied 3 eV and Method 12 applied 10 eV at ion funnel 1 (Table 1). LC-MS patterns displayed only slight changes, with Method 4 yielding 7141 peaks, 869 clusters, and 346 groups, and Method 12 producing 6614 peaks, 769 clusters, and 296 groups (Table 2 and Figure 3). Protein annotation by top-down sequencing was also comparable, as will be described in the next chapter. 

#### 2.2.3. Charge State Preference Does Not Influence Precursor Selection

Methods 9 and 11 (IS-CID + CID) only differed in their charge state preferred range. No charge state was specified in Method 11 (Table 1). In Method 9, a preference for the most abundant ions bearing 7‒15 charges was applied to assess whether it would favour the selection of highly protonated ions for CID fragmentation. This parameter had no impact on the selection process of parent ions since both methods generated similar numbers of peaks, clusters and groups and, most importantly, an identical number of MS2 spectra (218) (Table 2). This indicated that the automatic MS2 mode of the Q-ToF aptly selected highly charged protein ions for subsequent CID fragmentation. 

#### 2.2.4. A Long Duty Cycle Minimises MS/MS Events

To maximise signal sensitivity and minimise background noise, high summation values were utilised, which resulted in duty cycles that lasted at least 2.7 s (MS1 only—Method 1) (Table 2). Furthermore, these duty cycles were extended upon MS/MS analyses in a method-specific fashion (from 5.5 s in Methods 4 and 12, 13.7 s in Methods 2–3 and 5–6, to 24.6 s in Methods 9–11), which restricted the number of full MS1 scans (Table 2). Consequently, it limited how many ions could be selected as parents for the CID process during the LC run. 

Under our LC-MS/MS conditions, protein separation occurred from 2.5 to 25 min (22.5 min or 1350 s) with two to four precursors selected per duty cycle. The maximum number of MS2 events were computed and reported in Table 2. Apart from the CID-only methods, which only realised 28% (56/197 in Method 2) and 86% (170/198 in Method 3) of their potential due to high threshold requirements, all the other methods delivered 99–100% of the expected number of MS2 events (Table 2). However, these numbers are low. Instruments of high resolution offering a faster scanning capability would therefore be extremely advantageous. 

In our study, the slow scanning rate was partially alleviated by resorting to IS-CID only (Methods 4 and 12), whose fast fragmentation mode allowed for shorter duty cycles (5.5 s) and up to 247 scans during the 22.5-min separation (Table 2).

#### 2.2.5. High Energy Fragmentation Produces Lower *m*/*z* Ions

Overall, low-energy fragmentation was applied at the ion funnel 1 exit during the IS-CID MS mode (3, 5 or 10 eV), except for three methods for which high energy was applied. Methods 5–7 applied 30, 50, and 70 eV, respectively (Table 1). The consequences of high-energy IS-CID become visible upon reaching 70 eV (Method 7), which generates many more ions of less than 700 *m*/*z*, indicative of protein fragmentation. For instance, in the mixed standard sample, Method 1 (MS1 only) resulted in 8728 peaks, with the vast majority above 700 *m*/*z* (8711, 99.8%). Method 5 (30 eV) produced 4509 peaks in total with only 6 (0.1%) below 700 *m*/*z*. Conversely, Method 7 (70 eV) produced 3692 peaks in total, of which 93 (2.5%) were of less than 700 *m*/*z* (Table 2). 

Similarly, low-energy fragmentation (10 or 15 eV) was applied at the ion funnel 2 entrance during the IS-CID MS/MS mode, except for two methods for which greater energy was delivered. Methods 8 and 10 applied 20 and 30 eV, respectively (Table 1). Increasing the energy level during the IS-CID MS/MS step proved even more effective at fragmenting intact proteins within the ESI source, as more spectral peaks of low *m*/*z* were created (Figure 3). For instance, in the mixed standard sample, applying 20 eV (Method 8) or 30 eV (Method 10) resulted in the proportion of peaks below 700 *m*/*z* reaching 8.5% (281/3321 peaks) and 10.4% (188/1816 peaks) for Methods 8 and 10, respectively (Table 2). This resulted in ions of lower *m*/*z* being selected as precursors for a subsequent CID process. In the mixed standard sample, only three (1.5%) MS2 spectra resulted from precursors of less than 700 *m*/*z* using Method 5, while 20 (9.2%) MS2 spectra were acquired from precursors of less than 700 *m*/*z* using Method 10 (Table 2). However, as will be reported in the next chapter, such enhanced in-source fragmentation did not benefit the top-down sequencing output.

#### 2.2.6. CID Versus IS-CID

While CID parameters mostly revolve around ion features (e.g., intensity threshold, charge state, number of precursors) that allow for relatively controlled and robust conditions, IS-CID parameters themselves pertain to factors operating globally within the ESI source and the ion transfer region, the most important one being the DC voltage applied, which affects signal sensitivity. Owing to its less controllable nature, the IS-CID fragmentation process is more complex and far less predictable than MS/MS results. Therefore, a prerequisite of IS-CID efficacy is good chromatographic resolution with little or no background contamination [16]. 

Our ESI-Q-ToF mass spectrometer establishes IS-CID fragmentation not within the ESI source per se but more accurately within the ion transfer area called funnel 2. The two funnel-staged ion transfer region separates the ions from the drying gas and solvent, as well as transfers these ions, with minimal losses, to the quadrupole stage. The first and second funnel stages are separated by a DC plate. By increasing the DC potentials of funnel 1, the ions are accelerated into funnel 2, which in turn activates IS-CID. This ingenious, albeit unpredictable, fragmentation mode was invented 30 years ago and reported to favour highly charged species at lower energetic collision (i.e., cone voltage) akin to conventional CID process [5]. As with CID, as the ions’ *m*/*z* increase, the IS-CID efficacy decreases. That was confirmed in our experiment. Parent ions were predominantly sampled from the 800–1300 *m*/*z* range, where the most intense ions resolved, and only a handful of precursors arose from 1600 *m*/*z* and above (Table 2). 

IS-CID in combination with conventional CID on a ESI-Q-ToF system was tested at increasing cone voltage conditions (from 45 to 90 V) by Ginter and colleagues (2004) to top-down sequence a mixture of seven protein standards; they reported that some proteins necessitated higher voltage than others for in-source fragmentation to occur [28]. In a different study where only IS-CID was applied to generate MS2 spectra using a ESI-Q-ToF instrument, source cone voltage varied from 20 to 70 V and was evaluated on a sample mixture of 13 protein standards [29]. The authors also concluded that IS-CID efficient fragmentation greatly depended on cone voltage; in their experience, 40–60 V proved the most optimal range. 

### 2.3. Top-Down Sequencing of Milk Proteins

In the present study, a total of 11 MS/MS methods (Methods 2–12) were compared and the ultimate validation criterium was the coverage depth resulting from top-down sequencing annotation. The nine most abundant proteins known from milk were chosen to systematically assess AA sequence coverage across the 11 methods using Genedata Expressionist program. These proteoforms were α_S1_-CN B-8P, α_S2_-CN A-11P, β-CN A1, β-CN A2, κ-CN A-1P, κ-CN B-1P, α-LA B, β-LG A, and β-LG B. Table 3 documents the number of AAs top-down sequenced for each protein from either individual or mixed standards, or milk samples from Jerseys or Holsteins. 

Results were consistent from sample to sample, particularly for the proteins that responded very well to top-down sequencing such as β-CN A2 with a coefficient of variation (CV) as low as 0.4%. 

**Figure 4:**
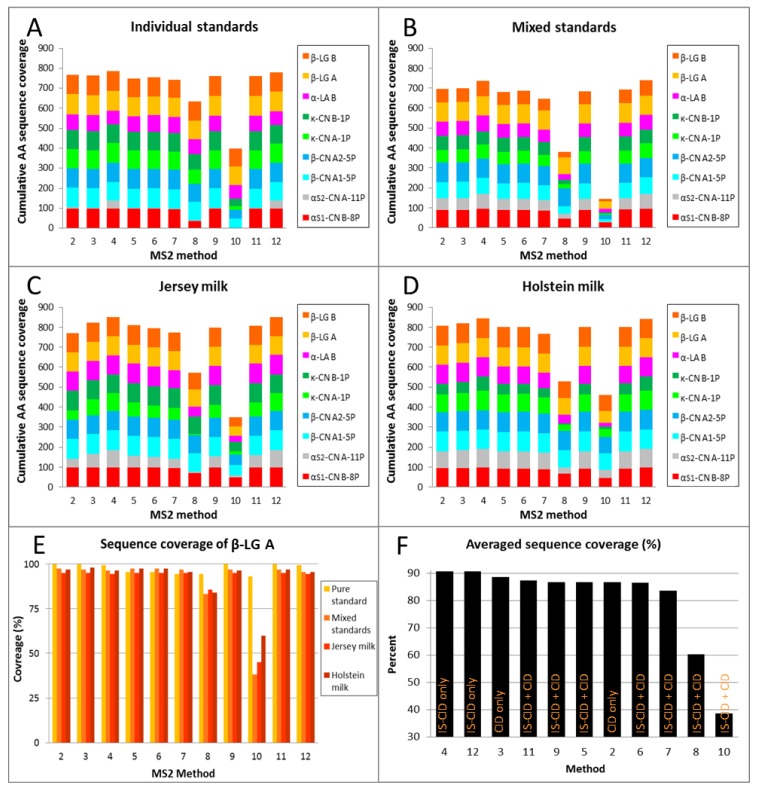
Comparison of the 11 MS/MS methods based on the AA sequence coverage in percent of the nine most prominent milk proteins. (**A**) Cumulative coverage in the individual standards. (**B**) Cumulative coverage in the mixed standards. (**C**) Cumulative coverage in the Jersey milk sample. (**D**) Cumulative coverage in the Holstein milk sample. (**E**) Illustration of AA sequence coverage for one particular protein, β-LG A, in increasingly complex samples. (**F**) Averaged sequence coverage for all eight proteins sorted by method efficacy; the fragmentation modes are reported as well.

For ease of interpretation, the values reported in Table 2 were converted to percentages and displayed as histograms (Figure 4). 

#### 2.3.1. Reproducibility and Protein Specificity

While being both protein- and method-specific, top-down sequencing results were reproducible across samples of increasing complexity, from the least (individual standards, Figure 4A) and mildly (mixed standards, Figure 4B) complex, to the most complex samples (raw milk, Figure 4C–D). The sample reproducibility was also demonstrated on one particular protein, β-LG A, which exhibited CV values spanning from 1.0% (Method 7) to 1.9% (Methods 9 and 12), omitting Methods 8 and 10, which were not only the least efficient but also the least reproducible (Figure 4E). As observed in previous studies [28,29], top-down sequencing success is protein-dependent. In our conditions, α_S2_-CN A-10P was the least responsive, possibly due to its elevated number of phosphorylation sites, while β-LGs and β-CNs were the most responsive. 

#### 2.3.2. Low-Energy Fragmentation Drives Top-Down Sequencing Efficacy

The histograms in Figure 4 also clearly demonstrate which methods were suitable for top-down sequencing and which were not. In Figure 4F, the methods have been sorted in descending order of AA sequence coverage. Under our conditions, the best methods were Methods 4 and 12 (90.6% sequence coverage on average), closely followed by Methods 3 (88.6%), 11 (87.4%), 9 (86.8%), 5 and 2 (86.7%), 6 (86.6%), and 7 (83.6%). The worst-performing methods were 8 (60.2%) and 10 (38.6%). Based on our observations, we can conclude that the methods that resorted to IS-CID on its own with little energy applied, in combination with CID or not, were best suited to top-down sequence prominent milk proteins. 

Optimal conditions in our case involved applying 3 eV (Method 4) or 10 eV (Method 12) to the ion funnel 1 exit during the IS-CID MS step and 15 eV to the ion funnel 2 entrance during the IS-CID MS/MS step. What seemed to be detrimental for our purposes was using too much energy (20 eV in Method 8 and 30 eV in Method 10) during the IS-CID MS/MS step. Moreover, we do not recommend applying an energy level higher than 10 eV during the IS-CID MS step, as was tested in Methods 5 (30 eV), 6 (50 eV), and 7 (70 eV). 

#### 2.3.3. AA Position Has No Influence on Fragmentation

In an attempt to further explain how CID-based fragmentation operated under our conditions, we aligned side by side all the AA sequences of the nine proteins of interest for each of the 11 MS/MS methods and blackened the AAs that were successfully top-down sequenced. For each protein, the sequenced AAs were counted across the methods and, as a result, we generated a score from 0 (not sequenced at all) to 11 (sequenced in all methods). Based on this scoring system, the corresponding position in the sequence was coloured red (the higher the score, the darker the shade). This is represented in Supplementary Figure S1. This display allowed for the quick visualisation of regions that remained unfragmented under our various conditions. Such recalcitrant regions were scattered along the protein sequence, suggesting that the AA position had no influence over fragmentation efficiency. 

All cow’s milk caseins are heavily phosphorylated. There was no visible pattern associated with phosphorylation sites (highlighted in yellow) either, apart from α_S2_-CN A-10P, for which five phosphorylated serine residues (S at positions 7, 13, 31, 56, and 143) resisted fragmentation. PTMs were not investigated in this study. Milk proteins are also glycosylated, as has been well documented for κ-CN, for instance [36]. Glycosylation sites could account for areas difficult to fragment, but we could not test this hypothesis in the present study. 

#### 2.3.4. Hydrophobicity Affects Fragmentation

To further elucidate whether AAs themselves influenced fragmentation efficiency, we converted the AA counts from Supplementary Figure S1 into percentages, sorted them according to their highest fragmentation efficiency (meaning sequenced across all 11 MS/MS methods) and plotted them (Supplementary Figure S2). Underneath the chart we listed the physical properties attributed to AAs (ThermoFisher Scientific website) to assist us in finding a pattern. 

In this representation, leucine, glycine, cysteine, tryptophan, histidine, and isoleucine displayed the best response to top-down sequencing with a success rate above 17% across 11 MS/MS methods (Supplementary Figure S2). These AAs are moderately to highly hydrophobic. Conversely, threonine, asparagine and arginine showed the lowest success rate (less than 9% across the 11 methods). Such AAs are hydrophilic. Our results suggest that AA hydrophobicity level has an impact on fragmentation efficiency.

#### 2.3.5. High Sequence Coverage Is Critical for Allelic Variants 

High sequence coverage is a prerequisite to successfully distinguishing between protein allelic variants such as β-CN A1 and A2, which only vary at position 67 (H to P); κ-CN A and B, which vary at positions 136 (T to I) and 148 (D to A); and β-LG A and B, which vary at positions 64 (D to G) and 118 (V to A). The mixed standard sample, Method 10, and, in the case of κ-CNs, Method 8 failed to deliver such relevant information since AAs at the aforementioned positions were not sequenced (Supplementary Figure S1). 

Interestingly, some genetic variants proved more amenable to top-down sequencing than others. For instance, in the mixed standard sample processed with Method 12, β-CN A2 (97%) was more thoroughly sequenced than β-CN A1 (83%). Likewise, β-LG A (96%) displayed greater sequence coverage than β-LG B (77%); this was also observed for κ-CN A (73%) and κ-CN B (68%) (Table 3 and Figure S1). In Holstein milk samples, we managed to reach 100% sequence coverage of β-LG B in six out of 11 MS/MS methods. Complete top-down sequencing was also achieved for β-LG A in individual standard samples in four MS/MS methods (Table 3). 

In the early days of top-down proteomics on ESI-Q-ToF platforms, the resulting short sequence tags from C- and N-termini from small to medium-sized proteins were just long enough to unambiguously identify the protein family, but not their allelic variants [26,27,28,29,30]. Almost complete top-down sequencing was achieved in 2009 with 17 kDa horse myoglobin and 30 kDa bovine carbonic anhydrase II being 92% and 96% sequenced, respectively [31]. Since then and to our knowledge, Q-ToF instruments have not been employed for top-down sequencing of intact proteins from complex biological matrices because they have been superseded by more expensive platforms such as FT mass spectrometers, offering better power resolution and alternative fragmentation methods like ETD, HCD, and ECD.

## 3. Materials and Methods 

### 3.1. Materials and Sample Preparation

The experimental design is schematised in Figure 1. Protein standards purchased from Sigma-Aldrich (Castle Hill, NSW, Australia) were alpha-casein (α-CN) from bovine milk (C6780-250MG, 70% pure), beta-casein (β-CN) from bovine milk (C6905-250MG, 98% pure), kappa-casein (κ-CN) from bovine milk (C0406-250MG, 70% pure), alpha-lactalbumin (α-LA) from bovine milk (L5385-25MG, 85% pure), and beta-lactoglobulin (β-LG) from bovine milk (L3908-250MG, 90% pure). These lyophilised standards were prepared as described in [23]. Briefly, they were fully solubilised at a 10 mg/mL concentration in 50% MilliQ (MerckMillipore, Bayswater, VIC, Australia) water/50% solution A (0.1 M Bis-Tris, 6 M Guanidine-HCl, 5.37 mM sodium citrate tribasic dehydrate, and 20 mM DTT). A volume of 50% acetic acid to reach 1% acetic acid final concentration was added to the standards. A 0.1-mL aliquot of the solubilised standard was transferred into a 100-μL glass insert placed in a 2-mL glass vial for immediate analysis by LC-MS. A standard mixture was prepared by mixing individual standards in the following proportions to account for various ionisation efficiency [23]: 25% α-CN, 25% κ-CN, 20% β-LG, 20% β-CN, and 10% α-LA.

Milk collection from Holstein-Friesian cows (coded H) and Jersey cows (coded J) was described in [35]. Milk sample preparation was described in [23]. Briefly, 0.5 mL of cold skim milk was transferred into a 1.5-mL tube and 0.5 mL of Solution A was added. A 0.02 mL volume of 50% acetic acid (1% acetic acid final concentration, pH 5.8) was then added. A 0.1-mL aliquot of the milk protein extract was transferred into a 100-μL glass insert placed in a 2-mL glass vial for immediate analysis by LC-MS.

### 3.2. HPLC Separation of Intact Proteins

The separation of intact proteins by UHPLC 1290 Infinity Binary LC system (Agilent) through a Aeris™ WIDEPORE XB-C8 (3.6 μm particle size, 200 Å pore size, 150 × 2.1 mm dimensions, C8 reverse phase core-shell silica from Phenomenex (Lane Cove, NSW, Australia) column at 75 °C was described in [23]. In brief, 3 μL of sample was injected and separated as followed: starting conditions 20% B, ramping to 28% B in 2.5 min, ramping to 40% B in 27.5 min, ramping to 99% B in 1 min and held for 4 min, lowering to 20% B in 0.1 min, equilibration at 20% B for 4.9 min. Mobile phase flow rates was 200 μL/min. Mobile phase A contained ACN with 0.1% FA and 0.02% trifluoroacetic acid (TFA), and mobile phase B contained H2O with 0.1% FA and 0.02% TFA. The diode array detector (DAD) spectrum was acquired from 190 to 400 nm. The pressure limit was set at 600 bars. 

### 3.3. MS1 Analysis

MS1 analysis was described in [23]. The UHPLC was on-line with a maXis HD UHR-Q-ToF (60,000 resolution) fitted with a standard ESI Apollo-source (BrukerDaltonikGmbh, Preston, VIC, Australia). To ensure mass accuracy, a Na-formate solution was infused continuously at 0.1 mL/h and the first 2.5 min of each run were used to re-calibrate masses post-acquisition. Each 40-min run was thus segmented as follows: 2.5 min to waste and the following 37.5 min to source. Capillary voltage was set at 4500 V. The nebuliser was set at 1.5 bar. The dry gas was set at 8 L/min. The dry temperature was set at 190 °C. The transfer funnel RF and multipole RF were set at 400 Vpp; no IS-CID energy was applied. The quadrupole ion energy was 5 eV, the collision cell energy was 10eV, and the collision RF 1800 Vpp. The ion cooler transfer time was 120 μs, with a prepulse storage of 10 μs and a RF of 400 Vpp. The ion polarity was positive and scan mode was MS. The rolling average mode was activated and set at 2. This MS1 method is called Method 1 hereafter.

### 3.4. MS2 Analyses and Top-Down Sequencing

MS/MS experiments were performed using the same LC parameters and MS1 parameters on the Q-TOF mass spectrometer as described above. A total of 11 MS2 methods were tested; their parameters are indicated in Table 1. These methods are referred to as Method 2 to Method 12 hereafter. LC-MS files were visualised using Bruker Compass DataAnalysis version 4.2. MS2 spectra were annotated using Bruker Biotools version 3.2 and SequenceEditor version 3.2. The retrieval of AA sequences was detailed in [23].

### 3.5. Protein Annotation in Genedata Expressionist

The data files obtained following LC-MS analysis using MS2 methods 2 to 12 were curated in the Refiner MS module of Genedata Expressionist^®^ version 11.0 with the following parameters: (1) Conversion of MS/MS to primary MS. (2) Chromatogram chemical noise subtraction using moving average algorithm and a five-scan window. (3) Spectrum smoothing using a moving average algorithm and a five-point *m*/*z* window. (4) Chromatogram peak detection using a five-scan summation window, a minimum peak size of four scans with boundaries merge strategy and five-point maximum merge distance, a curvature-based peak detection with 70% intensity threshold and inflection points boundary determination. (5) Chromatogram isotope clustering using a 0.2 min and 20 ppm tolerances, a peptide isotope shaping with 1‒8 protons, a 0.6 log-ration maximum distance, mono-isotopic computation, linear charge dependency with a minimum size ratio of 0.65. (6) Singleton filtering. (7) Peptide mapping with a 50 ppm tolerance, unspecific enzyme, three missed cleavage maximum, 10 AAs minimum length, and variable modifications, as explained hereafter. The peptide mapping was performed using a text file containing all 49 AA sequences in FASTA format of the bovine allelic variants of milk caseins, alpha-lactalbumin, and beta-lactoglobulin (Farrell et al., 2004) with the following variable modifications: pyro-Glu (N-term Q) and Phospho (ST). Some aspects of this workflow are illustrated in Figure S3.

Protein deconvolution was also performed on Method 1 files in the Refiner MS module of Genedata Expressionist^®^ version 11.0 with the following parameters. (1) Data sweep to remove UV data. (2) Chromatogram chemical noise subtraction using moving average algorithm, with a subtraction method, a 71 scan window, and 60% quantile. (3) Chromatogram lock mass using the Na-formate ion series. (4) Retention time range restriction from 4 to 25 min. (5) Intact protein activity using the harmonic suppression deconvolution method with 0.02 Da steps and 5-30 kDa masses. (6) Intensity thresholding using a clipping method with an intensity of 50. (7) Spectrum baseline subtraction using a 99% quantile and 30 kDa *m*/*z* window. (8) Chromatogram retention time alignment using a pairwise alignment-based tree scheme with 50-scan search interval and a gap penalty of 1. 

All MS1 and MS2 files are available from the stable public repository MassIVE at the following URL: http://massive.ucsd.edu/ProteoSAFe/datasets.jsp with the accession number MSV000082070. 

### 3.6. Validation and Computational Analyses

Top-down sequencing results were visualised and validated in Genedata Expressionist^®^ version 11.0 Refiner MS module within the Peptide Mapping activity and all its subsidiary tabs as well as using Bruker Biotools version 3.2 and SequenceEditor version 3.2, as exemplified in Figure S3.

Top-down sequencing annotations (including b- and y-ions series) for each of the nine proteins of interest in every sample and each of the 11 MS/MS methods were exported from Refiner MS module as .csv files. The .csv files contained the list of peptides identified for each protein along with the peptide AA lengths and their position in the protein sequence they matched to. The .csv files were imported into Microsoft^®^ Excel 2016 software for further processing. Coverage information was derived from the length and position of the identified peptides relative to their matching protein sequence to produce Table 3 and Figure 4. AA responsiveness to MS2 fragmentation was obtained using the positions of the first and last AA of the identified peptides. These analyses produced Figures S1 and S2.

## 4. Conclusions

In this experiment, we have fine-tuned IS-CID parameters with and without traditional CID using a LC-ESI-Q-ToF system in order to optimise the top-down sequencing of milk intact proteins. The most efficient methods utilised IS-CID on its own at low energy. Full (100%) sequence covering could be obtained on β-LGs, and overall enough coverage was achieved to distinguish between allelic proteoforms, varying only by one or two AAs. Such information is highly biologically relevant as alleles influence function and phenotypic features. In bovine milk, for instance, 12 allelic variants of beta-caseins have been identified across different breeds and populations [37]. The most common variants, A1 and A2, differ at amino acid position 67 with histidine in A1 and proline in A2 milk, as a result of a single nucleotide difference. One application of this research would be screening for the presence of the A1 variant in cow’s milk samples.

## Figures and Tables

**Table 1:** Parameters applied on the Q-TOF mass spectrometer for the 12 MS methods.

Mode	Step	Parameter	Method 1	Method 2	Method 3	Method 4	Method 5	Method 6	Method 7	Method 8	Method 9	Method 10	Method 11	Method 12
MS	Source	End plate offset	500 V	500 V	500 V	500 V	500 V	500 V	500 V	500 V	500 V	500 V	500 V	500 V
MS	Source	Capillary	4500 V	4500 V	4500 V	4500 V	4500 V	4500 V	4500 V	4500 V	4500 V	4500 V	4500 V	4500 V
MS	Source	Nebuliser	1.5 bar	1.5 bar	1.5 bar	1.5 bar	1.5 bar	1.5 bar	1.5 bar	1.5 bar	1.5 bar	1.5 bar	1.5 bar	1.5 bar
MS	Source	Dry gas	8 L/min	8 L/min	8 L/min	8 L/min	8 L/min	8 L/min	8 L/min	8 L/min	8 L/min	8 L/min	8 L/min	8 L/min
MS	Source	Temperature	190 °C	190 °C	190 °C	190 °C	190 °C	190 °C	190 °C	190 °C	190 °C	190 °C	190 °C	190 °C
MS	Source	Waste (min)	0–2.5	0–2.5	0–2.5	0–2.5	0–2.5	0–2.5	0–2.5	0–2.5	0–2.5	0–2.5	0–2.5	0–2.5
MS	Source	Source (min)	2.5–40	2.5–40	2.5–40	2.5–40	2.5–40	2.5–40	2.5–40	2.5–40	2.5–40	2.5–40	2.5–40	2.5–40
MS	Transfer	Funnel RF	400 Vpp	400 Vpp	400 Vpp	400 Vpp	400 Vpp	400 Vpp	400 Vpp	400 Vpp	400 Vpp	400 Vpp	400 Vpp	400 Vpp
MS	Transfer	IS-CID energy	0 eV	0 eV	0 eV	3 eV	30 eV	50 eV	70 eV	5 eV	0 eV	3 eV	0 eV	10 eV
MS	Transfer	Transfer multipole RF	400 Vpp	400 Vpp	400 Vpp	400 Vpp	400 Vpp	400 Vpp	400 Vpp	400 Vpp	400 Vpp	400 Vpp	400 Vpp	400 Vpp
MS	Quadrupole	Ion energy	5 eV	5 eV	5 eV	5 eV	5 eV	5 eV	5 eV	5 eV	5 eV	5 eV	5 eV	5 eV
MS	Quadrupole	Low mass	300 *m*/*z*	300 *m*/*z*	300 *m*/*z*	300 *m*/*z*	300 *m*/*z*	300 *m*/*z*	300 *m*/*z*	300 *m*/*z*	300 *m*/*z*	300 *m*/*z*	300 *m*/*z*	300 *m*/*z*
MS	Collision cell	Collision energy	10 eV	10 eV	10 eV	10 eV	10 eV	10 eV	10 eV	10 eV	10 eV	10 eV	10 eV	10 eV
MS	Collision cell	Collision RF (Vpp)	1800	1800	1800	1800	1800	1800	1800	1800	1800	1800	1800	1800
MS	Collision cell	Transfer time	120 μs	120 μs	120 μs	120 μs	120 μs	120 μs	120 μs	120 μs	120 μs	150 μs	120 μs	120 μs
MS	Collision cell	Pre-pulse storage	10 μs	10 μs	10 μs	10 μs	10 μs	10 μs	10 μs	10 μs	10 μs	15 μs	10 μs	10 μs
MS	MS1	Ion polarity	positive	positive	positive	positive	Positive	Positive	positive	positive	positive	positive	positive	positive
MS	MS1	Mass range (*m/z*)	600–3000	600–3000	600–3000	200–2000	200–2000	200–2000	200–2000	200–2000	200–2000	200–2000	200–2000	200–2000
MS	MS1	Summation	15,000	15,000	15,000	18,321	18,321	18,321	18,321	18,321	18,321	18,321	18,321	18,321
MS	MS1	Rolling average mode	2	2	2	2	2	2	2	2	2	2	2	2
in source	IS-CID	IS-CID	NO	NO	NO	YES	YES	YES	YES	YES	YES	YES	YES	YES
in source	IS-CID	IS-CID MS (funnel 1 exit)				3 eV	30 eV	50 eV	70 eV	5 eV	3 eV	3 eV	3 eV	10 eV
in source	IS-CID	IS-CID MS/MS (funnel 2 entry)				15 eV	10 eV	10 eV	10 eV	20 eV	10 eV	30 eV	10 eV	15 eV
in source	IS-CID	Acquisition time factor				1	1	1	1	1	1	1	1	1
MS/MS	MS2	Auto MS/MS mode	OFF	ON	ON	OFF	ON	ON	ON	ON	ON	ON	ON	OFF
MS/MS	CID	Acquisition	NO	YES	YES	NO	YES	YES	YES	YES	YES	YES	YES	NO
MS/MS	CID	MS/MS low (per 1000 sum.)		10^4^ cts	10^4^ cts		10^4^ cts	10^4^ cts	10^4^ cts	10^4^ cts	10^4^ cts	10^4^ cts	10^4^ cts	
MS/MS	CID	MS/MS high (per 1000 sum.)		10^6^ cts	10^6^ cts		10^6^ cts	10^6^ cts	10^6^ cts	10^6^ cts	10^6^ cts	10^6^ cts	10^6^ cts	
MS/MS	MS2	Preference activated	NO	NO	NO	NO	NO	NO	NO	NO	YES	NO	NO	NO
MS/MS	MS2	Charge state preferred range									7–15			
MS/MS	MS2	Exclude singly									YES			
MS/MS	Precursor ions	Exclude (*m/z*)		100–600	100–600		100–600	100–600	100–600	100–600	100–600	100–600	100–600	
MS/MS	Precursor ions	No. of precursors		2	2		2	2	2	3	4	4	4	
MS/MS	Threshold	Absolute (per 1000 sum.)		1153 cts	170 cts		10 cts	10 cts	10 cts	10 cts	10 cts	10 cts	10 cts	
MS/MS	Threshold	Absolute		17,302 cts	2550 cts		183 cts	183 cts	183 cts	183 cts	183 cts	183 cts	183 cts	
MS/MS	Active exclusion	ON/OFF		ON	ON		ON	ON	ON	ON	ON	ON	ON	
MS/MS	Active exclusion	Exclude after		3 spectra	3 spectra		1 spectra	1 spectra	1 spectra	1 spectra	1 spectra	1 spectra	1 spectra	
MS/MS	Active exclusion	Release after		5 min	5 min		5 min	5 min	5 min	5 min	5 min	5 min	5 min	

**Table 2:** Number of spectral peaks, clusters, groups and MS/MS spectra found in the mixed standard sample across all 12 methods using Genedata Refiner workflow.

Method	1	2	3	4	5	6	7	8	9	10	11	12
LC separation (min)	22.5	22.5	22.5	22.5	22.5	22.5	22.5	22.5	22.5	22.5	22.5	22.5
LC separation (s)	1350	1350	1350	1350	1350	1350	1350	1350	1350	1350	1350	1350
Duty cycle (s)	2.7	13.7	13.7	5.6	13.7	13.7	13.8	19.1	24.6	24.6	24.6	5.5
No. of full MS1 scans	493	99	99	243	99	99	98	71	55	55	55	247
No. of precursors		2	2		2	2	2	3	4	4	4	
(a) Theor. no. of MS2 events	0	197	198	0	197	198	196	212	219	219	220	0
(b) MS1 peaks (300–3000 *m/z*)	8728	8402	5184	7141	4509	4415	3692	3321	3171	1816	3115	6614
(c) MS1 peaks (700–3000 *m/z*)	8711	8387	5184	7124	4503	4415	3599	3040	3169	1628	3113	6599
(d) MS1 peaks (300–700 *m/z*)	17	15	0	17	6	0	93	281	2	188	2	15
(d)/(b) (%)	0.2	0.2	0.0	0.2	0.1	0.0	2.5	8.5	0.1	10.4	0.1	0.2
MS1 clusters	1069	1044	601	869	551	536	530	654	386	405	371	769
MS1 groups	424	426	240	346	234	231	282	487	170	319	159	296
(e) Observed No. of MS2 spectra	0	56	170	0	196	196	196	210	218	218	218	0
(e)/(a) (%)		28	86		99	99	100	99	99	99	99	
(e)/(b) (%)		0.7	3.3		4.3	4.4	5.3	6.3	6.9	12.0	7.0	
MS2 *m/z* range		730–1636	644–1854		643–1955	703–1955	657–1966	609–1913	607–1774	609–1966	634–1928	
600–700 *m/z*			1		3		2	18	3	20	4	
700–800 *m/z*		2	2		5	5	5	14	4	19	8	
800–900 *m/z*		4	12		20	20	15	22	26	27	29	
900–1000 *m/z*		20	53		45	44	21	34	56	36	54	
1000–1100 *m/z*		28	77		53	51	47	23	63	24	57	
1100–1200 *m/z*			5		29	26	35	24	30	18	23	
1200–1300 *m/z*			2		19	21	26	26	20	29	18	
1300–1400 *m/z*			2		3	7	17	14	8	7	2	
1400–1500 *m/z*			2		2	5	4	11	2	10	5	
1500–1600 *m/z*			2		3	4	5	8	3	9	3	
1600–1700 *m/z*		2	6		4	4	7	7	1	8	7	
1700–1800 *m/z*			3		4	3	3	1	2	5	5	
1800–1900 *m/z*			3		4	4	7	7		4	1	
1900–2000 *m/z*					2	2	2	1		2	2	

**Table 3:** Number of AAs top-down sequenced across the 11 MS2 methods from the nine most abundant milk proteins with the individual standards, mixed standards, and milk samples.

Protein	Variant	PTM	Code	Mo. Mass (Da)	Length (AAs)	RT (min)	Sample 1	Method 2	Method 3	Method 4	Method 5	Method 6	Method 7	Method 8	Method 9	Method 10	Method 11	Method 12
α_S1_-CN	B	8P	α_S1_-CN B 8P	23,600.3	199	15–18	I	196	196	195	194	195	190	70	195	7	194	195
α_S2_-CN	A	11P	α_S2_-CN A 11P	25,213.0	207	6.8–8.3	I	14	6	83	6	6	6	6	6	0	6	83
β-CN	A1	5P	β-CN A1 5P	24,008.2	209	21–22	I	204	204	197	205	205	203	197	205	95	205	195
β-CN	A2	5P	β-CN A2 5P	23,968.2	209	21.5–23	I	202	202	201	203	203	201	186	203	92	203	200
κ-CN	A	1P	κ-CN A 1P	19,026.5	169	6.5–8.5	I	160	160	165	157	157	156	119	157	31	157	165
κ-CN	B	1P	κ-CN B 1P	18,993.6	169	8.5–10	I	162	162	157	160	159	157	133	161	59	161	154
α-LA	B		α-LA B	14,176.8	123	15–18	I	98	97	84	94	100	98	90	97	86	97	83
β-LG	A		β-LG A	18,355.5	162	22–24	I	162	162	161	155	155	153	153	162	151	162	161
β-LG	B		β-LG B	18,269.4	162	20–22	I	159	158	159	153	154	149	155	159	146	159	158
α_S1_-CN	B	8P	α_S1_-CN B 8P	23,600.3	199	15–18	M	178	178	192	179	177	173	91	178	52	181	192
α_S2_-CN	A	11P	α_S2_-CN A 11P	25,213.0	207	6.8–8.3	M	121	123	150	111	116	108	52	115	8	115	151
β-CN	A1	5P	β-CN A1 5P	24,008.2	209	21–22	M	171	172	168	162	167	156	79	163	27	165	174
β-CN	A2	5P	β-CN A2 5P	23,968.2	209	21.5–23	M	201	201	201	203	201	200	182	202	43	203	201
κ-CN	A	1P	κ-CN A 1P	19,026.5	169	6.5–8.5	M	108	109	122	106	109	93	41	106	14	107	124
κ-CN	B	1P	κ-CN B 1P	18,993.6	169	8.5–10	M	122	125	112	120	120	110	33	122	10	121	116
α-LA	B		α-LA B	14,176.8	123	15–18	M	86	85	96	83	82	77	37	83	21	86	94
β-LG	A		β-LG A	18,355.5	162	22–24	M	158	157	156	158	158	157	135	157	62	157	155
β-LG	B		β-LG B	18,269.4	162	20–22	M	111	112	126	106	107	96	42	106	20	111	125
α_S1_-CN	B	8P	α_S1_-CN B 8P	23,600.3	199	15–18	J	194	195	198	195	195	189	137	194	97	195	198
α_S2_-CN	A	11P	α_S2_-CN A 11P	25,213.0	207	6.8–8.3	J	92	140	177	122	110	97	18	114	20	126	177
β-CN	A1	5P	β-CN A1 5P	24,008.2	209	21–22	J	206	206	205	206	206	205	191	206	110	206	204
β-CN	A2	5P	β-CN A2 5P	23,968.2	209	21.5–23	J	199	199	201	200	200	199	188	199	106	199	201
κ-CN	A	1P	κ-CN A 1P	19,026.5	169	6.5–8.5	J	82	132	151	121	107	104	12	110	30	119	154
κ-CN	B	1P	κ-CN B 1P	18,993.6	169	8.5–10	J	164	164	157	164	163	164	146	164	78	163	157
α-LA	B		α-LA B	14,176.8	123	15–18	J	120	120	121	120	120	111	62	120	38	120	121
β-LG	A		β-LG A	18,355.5	162	22–24	J	154	154	153	154	154	154	139	154	73	154	153
β-LG	B		β-LG B	18,269.4	162	20–22	J	158	156	156	158	156	154	134	156	76	156	155
α_S1_-CN	B	8P	α_S1_-CN B 8P	23,600.3	199	15–18	H	191	191	197	183	184	177	133	185	89	183	197
α_S2_-CN	A	11P	α_S2_-CN A 11P	25,213.0	207	6.8–8.3	H	172	181	192	179	179	172	65	178	88	180	193
β-CN	A1	5P	β-CN A1 5P	24,008.2	209	21–22	H	204	204	200	204	204	204	181	204	170	204	201
β-CN	A2	5P	β-CN A2 5P	23,968.2	209	21.5–23	H	206	207	203	207	207	207	198	207	168	207	203
κ-CN	A	1P	κ-CN A 1P	19,026.5	169	6.5–8.5	H	147	150	165	147	153	133	54	151	72	150	165
κ-CN	B	1P	κ-CN B 1P	18,993.6	169	8.5–10	H	89	97	118	91	86	82	11	89	17	95	118
α-LA	B		α-LA B	14,176.8	123	15–18	H	118	119	119	106	105	94	52	107	23	104	119
β-LG	A		β-LG A	18,355.5	162	22–24	H	157	159	156	158	158	155	136	156	97	157	155
β-LG	B		β-LG B	18,269.4	162	20–22	H	162	162	159	162	162	160	138	162	128	162	158

I, individual standard; M, mixed standard; J, Jersey milk; H, Holstein milk.

## Supplementary Materials

The following are available online, Figure S1: Top-down sequencing responsiveness of AAs for each of the eight most abundant proteins from the mixed standard sample, Figure S2: CID-friendly amino acids from top-down sequenced milk proteins, Figure S3: Data processing of MS/MS files using Genedata Refiner and DataAnalysis/Biotools exemplified on the Holstein milk sample processed using Method 3.

## Abbreviations

**Abbreviation**	**Definition**
AA	Amino acid
ACN	Acetonitrile
AE	Active exclusion
CAD	Collisionally activated dissociation
CID	Collision-induced dissociation
CV	Coefficient of variation
DAD	Diode array detector
DC	Direct current
ECD	Electron capture dissociation
ESI	Electrospray ionisation
ETD	Electron transfer dissociation
eV	Electron-volt
FA	Formic acid
FT	Fourier transform
HCD	Higher-energy collisional dissociation
HPLC	High-performance liquid chromatography
IS-CID	In-source CID
kDa	KiloDalton
keV	Kiloelectron-volt
LC-MS	Liquid chromatography‒mass spectrometry
LIT	Linear ion trap
*m/z*	Mass-to-charge ratio
MS	Mass spectrometry
MS/MS	Tandem MS
MS1	Full scan MS
MS2	Tandem MS
MW	Molecular weight
ppm	Parts per million
PTM	Post-translational modification
Q	Quadrupole
QQQ	Triple quadrupole
Q-ToF	Quadrupole time-of-flight
RF	Radiofrequency
TFA	Trifluoroacetic acid
ToF	Time-of-flight
UHT	Ultra-high temperature
V	Volt
α-LA	Alpha-lactalbumin
αS1-CN	Alpha-S1-casein
αS2-CN	Alpha-S2-casein
β-CN	Beta-casein
β-LG	Beta-lactoglobulin
κ-CN	Kappa-casein
