# Supplementary material for: Optimisation of Milk Protein Top-Down Sequencing Using In-Source Collision-Induced Dissociation in the Maxis Quadrupole Time-of-Flight Mass Spectrometer
Source: Molecules. 2018 Oct 26;23(11):2777. doi: 10.3390/molecules23112777 (PMC6278275; doi:10.3390/molecules23112777)

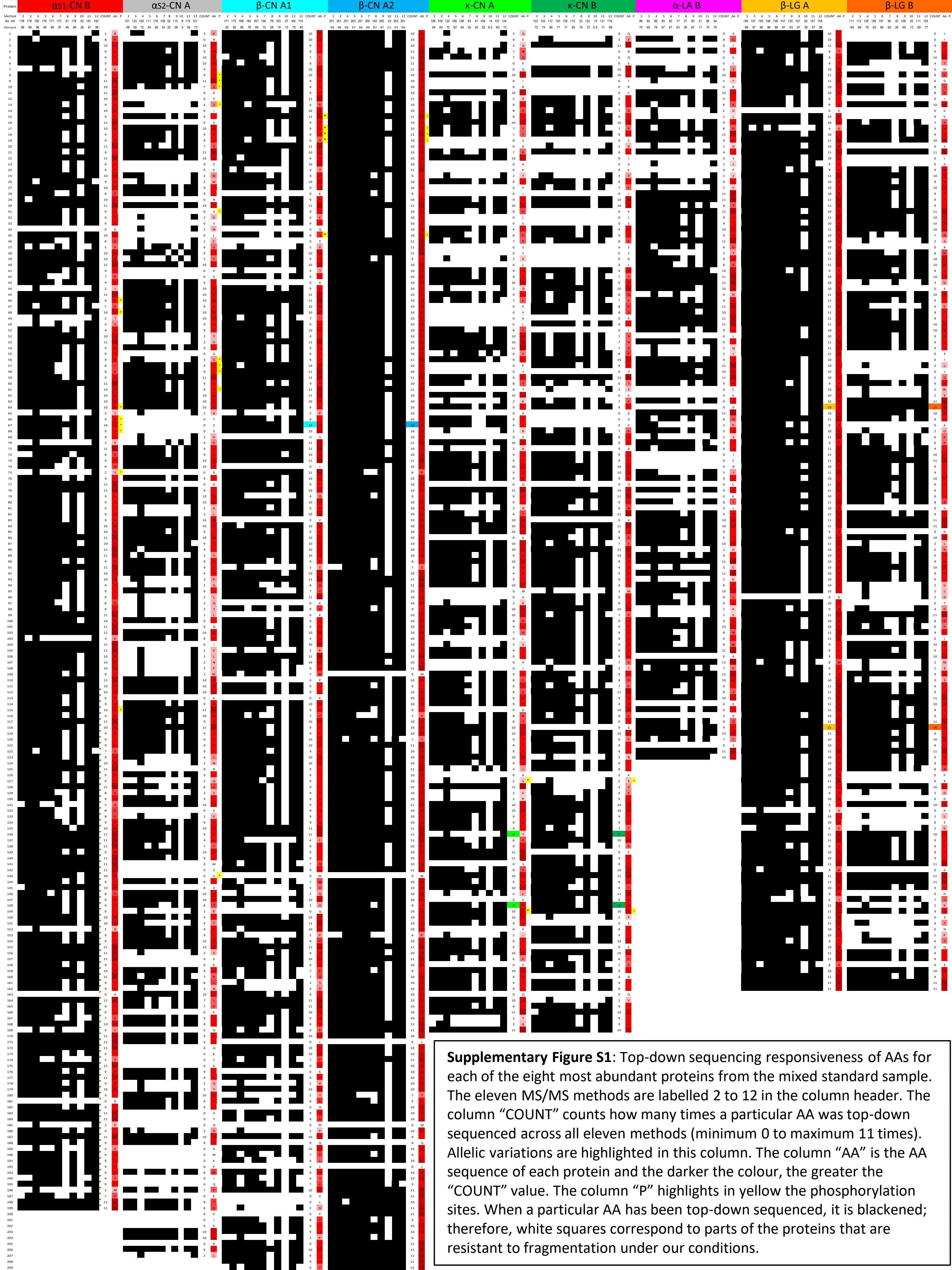

Supplementary Figure S1: Top-down sequencing responsiveness of AAs for each of the eight most abundant proteins from the mixed standard sample. The eleven MS/MS methods are labelled 2 to 12 in the column header. The column "COUNT" counts how many times a particular AA was top-down sequenced across all eleven methods (minimum 0 to maximum 11 times). Allelic variations are highlighted in this column. The column "AA" is the AA sequence of each protein and the darker the colour, the greater the "COUNT" value. The column "P" highlights in yellow the phosphorylation sites. When a particular AA has been top-down sequenced, it is blackened; therefore, white squares correspond to parts of the proteins that are resistant to fragmentation under our conditions.

Supplementary Figure S2: CID-friendly amino acids from top-down sequenced milk proteins. Using the data of all eight proteins presented in Suppl. Fig. S1 and the AAs that were successfully fragmented in all eleven MS/MS methods (darkest green) as a reference, all twenty AAs were sorted in a descending fashion based on their “COUNT” values. The lower panel lists some known features of AAs. It seems hydrophic AAs are more amenable to fragmentation than hydrophilic AAs under our conditions.

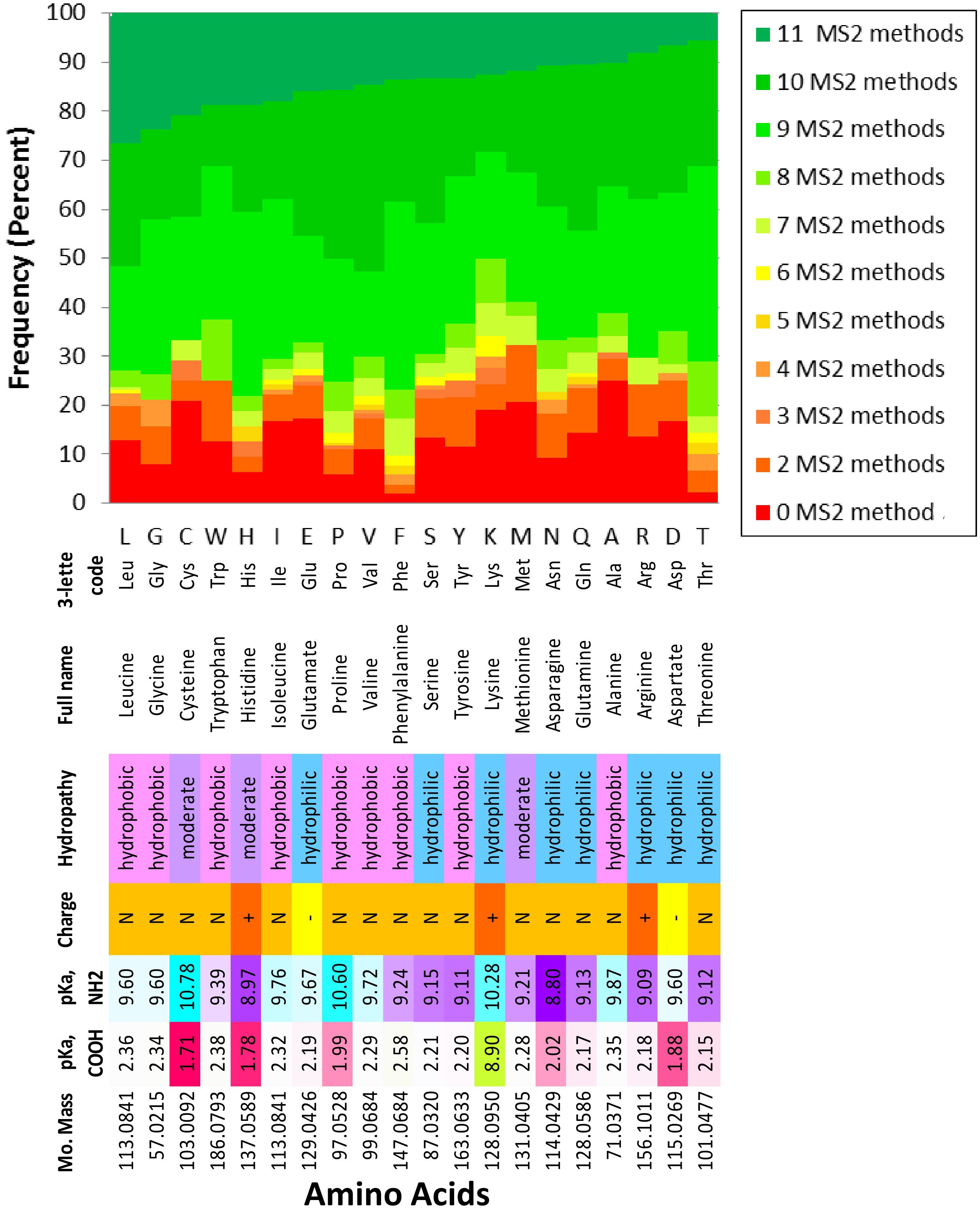

	L	G	C	W	H	I	E	P	V	F	S	Y	K	M	N	Q	A	R	D	T
3-lette code	Leu	Gly	Cys	Trp	His	Ile	Glu	Pro	Val	Phe	Ser	Tyr	Lys	Met	Asn	Gln	Ala	Arg	Asp	Thr
Full name	Leucine	Glycine	Cysteine	Tryptophan	Histidine	Isoleucine	Glutamate	Proline	Valine	Phenylalanine	Serine	Tyrosine	Lysine	Methionine	Asparagine	Glutamine	Alanine	Arginine	Aspartate	Threonine
Hydropathy	hydrophobic	hydrophobic	moderate	hydrophobic	moderate	hydrophobic	hydrophilic	hydrophobic	hydrophobic	hydrophobic	hydrophilic	hydrophobic	hydrophilic	moderate	hydrophilic	hydrophilic	hydrophobic	hydrophilic	hydrophilic	hydrophilic
Charge	N	N	N	N	+	N	-	N	N	N	N	N	+	N	N	N	N	+	-	N
pKa, NH2	9.60	9.60	10.78	9.39	8.97	9.76	9.67	10.60	9.72	9.24	9.15	9.11	10.28	9.21	8.80	9.13	9.87	9.09	9.60	9.12
pKa, COOH	2.36	2.34	1.71	2.38	1.78	2.32	2.19	1.99	2.29	2.58	2.21	2.20	8.90	2.28	2.02	2.17	2.35	2.18	1.88	2.15
Mo. Mass	113.0841	57.0215	103.0092	186.0793	137.0589	113.0841	129.0426	97.0528	99.0684	147.0684	87.0320	163.0633	128.0950	131.0405	114.0429	128.0586	71.0371	156.1011	115.0269	101.0477

Amino Acids

Supplementary Figure S3: Data processing of MS/MS files using Genedata Refiner and DataAnalysis/Biotools exemplified on the Holstein milk sample subject to method 3. **A**. Key steps in the Genedata Refiner workflow with zoomed-in depictions of the raw data, the data following smoothing and noise removal, peak detection, peak clustering, and cluster grouped. Boxed areas on the upper panel are further zoomed-in on the lower panel to illustrate isotopic resolution. Yellow arrows on the panel on the right hand corner indicates clusters that have been grouped together. Circles on the lower panel highlight the ion precursor selected for CID fragmentation. **B**. Full LC/MS map visualised in Genedata Refiner with *m/z* on the x axis and LC retention time in min on the y axis following smoothing and noise removal. The boxed area corresponds to the zoom-in area of A. **C**. Full LC/MS map visualised in Genedata Refiner with *m/z* on the x axis and LC retention time in min on the y axis cluster grouping. The boxed area corresponds cluster group of β-LG B. White dots represent true MS/MS (MS2) events using conventional CID fragmentation. **D**. Full MS1 spectra at 21.4 min and MS2 spectra resulting from CID fragmentation of 914.7364 *m/z* parent ion visualised in DataAnalysis (Bruker) sent to Biotools (Bruker) for b- and y-type ion annotation using β-LG B sequence. **E**. Top-down sequencing results of β-LG B visualised in Genedata Refiner which illustrates the ladder fragmentation pattern.

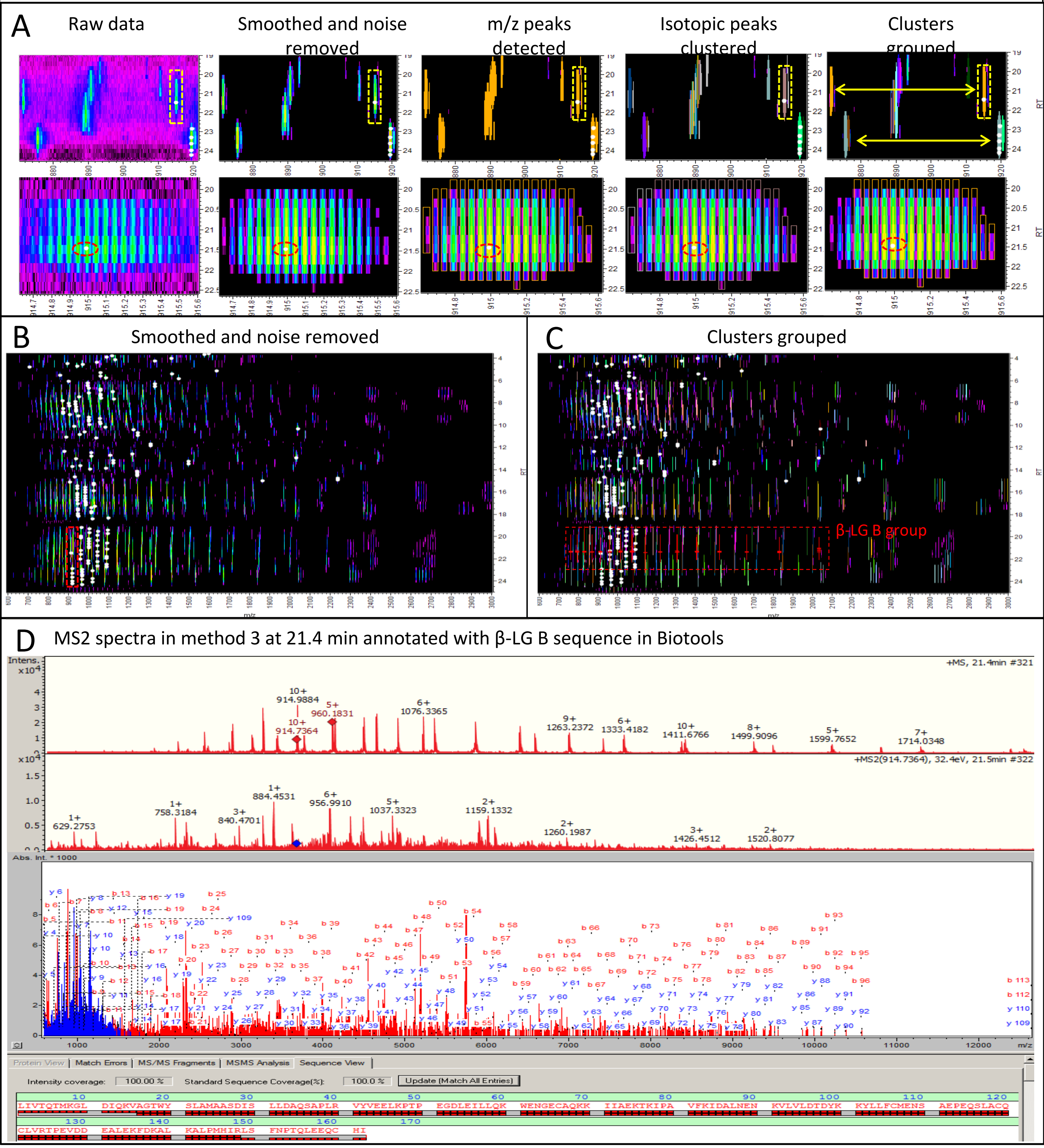

E Top-down sequencing of β-LG B sequence (162 AAs) in method 3 (100% coverage)

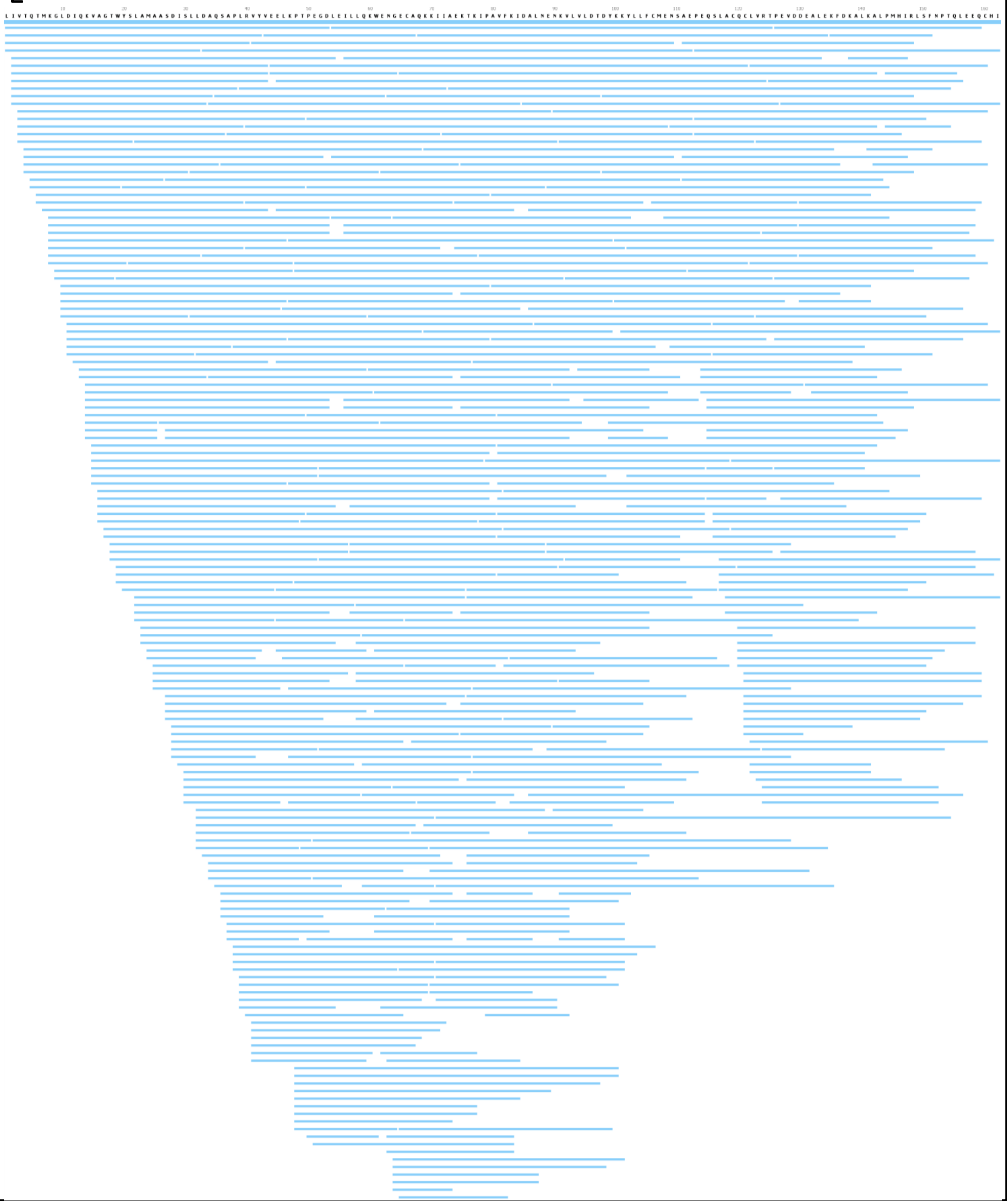

Supplement: Supplementary file 1 [file molecules-23-02777-s001.pdf]
